# Suggestion

**Published:** 1863-07

**Authors:** W. C. Duncan


### A SUGGESTION.

We are indebted to Drs. Lewis & Son, of Buffalo, for the following improvement in the Hayes' vulcanizing oven; designed to prevent the opening of the flask while vulcanizing. The flask for this purpose should be one of the new pattern, with a groove down each side to admit of the passage of a stirrup over it. Two holes should be drilled through the casting forming the bottom of the vulcanizer, to correspond with the grooves in the flask, so that a stirrup of "quarter inch" round iron can be placed over the flask when it is in its proper position in the vulcanizer; the ends of the stirrup extending through the bottom of the vulcanizer sufficiently far for the attachment of nuts. These nuts should be faced in a lathe and the surface they bear against should be counterbored, so that it shall be true and smooth. If the work is well done, a small ring cut from a rubber tube, and slipped on the stirrup before the nut is put on, will be sufficient to make a steam tight joint. A couple of turns of cotton wicking will answer the same purpose. The nuts and holes should be *very slightly* countersunk; just enough to take off the corners. The same apparatus may be used for bringing the flask together after it is packed, instead of the "spring clamp." The spring bail may be removed, and a couple of holes drilled through the bottom casting as before. *

---

Mr. Editor:—The following I am satisfied will be useful to many Dentists:

To separate teeth from a rubber base and preserve them unmarred, take chemically pure nitric acid and put the work into it, using for the purpose a glass vessel, and in one hour the teeth will come out looking as fresh as if from the hands of the manufacturer. I have practiced the mode for some-time with the best of success. Yours Truly,

W. C. Duncan.

